# Editorial: Advances in extracorporeal life support in critically ill patients, volume II

**DOI:** 10.3389/fmed.2023.1130962

**Published:** 2023-01-19

**Authors:** Guo-wei Tu, Yih-Sharng Chen, Man Huang, Nikola Dobrilovic, Zhe Luo

**Affiliations:** ^1^Department of Critical Care Medicine, Zhongshan Hospital, Fudan University, Shanghai, China; ^2^Department of Cardiovascular Surgery, National Taiwan University Hospital, Taipei, Taiwan; ^3^Department of Intensive Care Unit, Second Affiliated Hospital, School of Medicine, Zhejiang University, Hangzhou, China; ^4^Division of Cardiac Surgery, NorthShore University HealthSystem, Chicago, IL, United States; ^5^Department of Critical Care Medicine, Zhongshan Hospital (Xiamen), Fudan University, Xiamen, China

**Keywords:** Extracorporeal Membrane Oxygenation, transportation, transplantation, Intra-Aortic Balloon Pump, complication

Initially used as a salvage strategy for patients with refractory cardiac and/or respiratory failure, Extracorporeal Membrane Oxygenation (ECMO) has grown in popularity since its successful application outside the operating room in the 1970's, and this trend is particularly evident in the current years (1–4). In addition, the number of centers able to provide ECMO support has expanded drastically with evolving technology and simplifying procedures (3). Therefore, the Research Topic of indications, procedures, management, and even transport of ECMO patients will never be out of date.

In this Research Topic, we were able to collect volume II of advances in extracorporeal life support in critically ill patients (https://www.frontiersin.org/research-topics/33279/advances-in-extracorporeal-life-support-in-critically-ill-patients-volume-ii). The topic comprises 17 papers in this Research Topic, of which 12 are original researches and five are case reports, encompassing indications, procedure, management and complications of extracorporeal life support.

As a salvage therapy, ECMO can be life-saving in numerous clinical scenarios (5, 6). In this volume, Xu et al. reported a case of a 14-year-old adolescent male patient with an anomalous left coronary artery originating from the right coronary sinus rescued by VA-ECMO and Intra-Aortic Balloon Pump followed. In addition, ECMO are often used as a supportive technique to perform surgeries in all kinds of emergent conditions (7). Zhang et al. reported a case of a human immunodeficiency virus patient with severe lower tracheal obstruction underwent rigid bronchoscopy, airway tumor resection and Y-type silicone stent with ECMO supported. While Tian et al. (b) reported a case of ECMO allowing AngioJet thrombectomy in a patient with severe multiple trauma and acute massive pulmonary embolism. Nowadays, VA-ECMO is being increasingly performed by the percutaneous technique, usually under ultrasound guidance (8, 9). Correspondingly, percutaneous decannulation of VA-ECMO in these patients is thereby receiving growing concerns (9–11). In this volume, Tian et al. (a) reported a case of successfully decannulation of VA-ECMO with Perclose ProGlide device application and achieved total percutaneous post-closure of femoral arteriotomies. Except for ECMO, this volume also collected a case of hemophagocytic lymphohistiocytosis secondary to NK-type non-Hodgkin lymphoma and Epstein-Barr virus reactivation, which was presented with multiorgan dysfunction and distributive shock as the main manifestation, and was successfully treated by cytokine hemoadsorption.

In recent years, prophylactic use of ECMO in the intraoperative period has been increasingly reported. Zhao Y. et al. conducted a single center, retrospective study to evaluate the clinical outcomes and complications in lung transplantation recipients receiving intraoperative ECMO support, and found that patients received prophylactic ECMO support exhibited better survival and acceptable complication rates. Bai et al. assessed the value of prophylactic use of VA-ECMO in high-risk percutaneous coronary intervention. The consequence indicated that in complex and high-risk coronary artery lesions, ECMO utilization helps to attain safe and feasible revascularization.

Currently, it is common to transport ECMO patients to large referral centers for further treatment. In this volume, Zhao Y. C. et al. brought a retrospective analysis of 126 ECMO patients transferred from regional hospitals to large medical centers, finding no deaths reported though few life-threatening complications occurred during transport, and implying that transferring ECMO patients is feasible as long as careful evaluation and adequate preparation has conducted.

The effectiveness of life support relies on well-timed interventions, which requires moment-by-moment access to key information relevant to the technique adopted. Teng et al. evaluated the concordance between one commercial point-of-care activated partial thromboplastin time (POC-APTT) instrument and the laboratory APTT test in adult patients underwent ECMO support while accepting anticoagulation with unfractionated heparin. Although the consistency was weak and the commercial instrument evaluation was not fungible with the laboratory APTT test, the idea of POC assessing APTT is worth encouraging. Shi et al. explored the value of a multimodal neuromonitoring (MNM) protocol that holds promise for timely detection of neurological injury in VA-ECMO-supported patients. It turned out that the protocol can help identify and treat latent neurological injury in a timely manner, and ultimately improve long-term neurological outcomes. Cousin et al. evaluated carboxyhemoglobin as a novel marker for the incidence of ECMO oxygenator dysfunction. As a surrogate for free hemoglobin, carboxyhemoglobin demonstrated a better response.

Some inherent concerns of ECMO support, such as recirculation for VV-ECMO and vascular complications for VA-ECMO, were all covered in this volume. Fisser, Palmér et al. revealed that femoro-jugular configuration causes less recirculation by comparing recirculation fraction between femoro-jugular and jugulo-femoral VV-ECMO configurations. Also, the team identified risk factors for higher recirculation fraction including excessively short distance between cannula tips, higher ECMO flow, and lower heart rate. Hu et al. retrospectively analyzed the data from 179 adult patients underwent VA-ECMO, and drew a conclusion that for limb ischemia prediction, diabetes, application concomitant Intra-Aortic Balloon Pump, and peak vasoactive-inotropic score are independent risk factors. Fisser, Armbrüster et al. investigated the prevalence data on arterial and venous vascular complications among patients requiring VA-ECMO, and concluded the high incidence of the conditions, implying that screening for vascular complications and accepting anticoagulation should be implemented in such patients.

Early identification of ECMO patients with unsatisfactory prognosis would prompt early intervention and thus might make a change to the outcome. Huang et al. reported that after ECMO initiation, there is a great correlation between the serum total bilirubin and survival, as the risk of 28-day mortality remarkably elevated if hyperbilirubinemia occurs. Thus, the team recommended serum total bilirubin as a predictor for both its effect and its convenience to measure. Jin et al. retrospectively analyzed data collected from 101 pediatric patients receiving VA-ECMO to identify the risk factors of in-hospital death, and to validate the reliability of the current scoring system, including the Pediatric Extracorporeal Membrane Oxygenation Prediction (PEP) model, Pre-cannulation Pediatric Survival After VA-ECMO (Pedi-SAVE) score, and Post-cannulation Pedi-SAVE score. They found that lactate level and infectious complications before and during the ECMO application respectively were in-hospital mortality risk factors. Also, The pre-ECMO PEP score and the post-cannulation Pedi-SAVE score performed a high predictive capacity for in-hospital mortality in children patients underwent post-cardiotomy VA-ECMO.

In summary, this Research Topic compiled a series of cases and research articles that are related to ECMO support. We appreciate the work of all authors, reviewers, and editors for this volume, and we do believe that this volume will provide readers with new insights and inspiration for future research.

## Author contributions

G-wT drafted the manuscript. Y-SC, MH, ND, and ZL edited the manuscript, contributed to the Research Topic, and approved the publication of this editorial. All authors contributed to the article and approved the submitted version.

## References

[1] Bartlett RH Gazzaniga AB Wetmore N Mullin P Roohk V Haiduc N. Extracorporeal membrane oxygenator support for cardiopulmonary failure. Experience in 28 cases. J Thorac Cardiovasc Surg. (1977) 73:375–86. 10.1016/S0022-5223(19)39916-7 839827

[2] Bartlett RH Gazzaniga AB Wetmore N Mullin P Roohk V Haiduc N. Extracorporeal membrane oxygenator support for cardiopulmonary failure: Experience in 40 cases. Nihon Kyobu Geka Gakkai Zasshi. (1978) 26:249–63. 701862

[3] Thiagarajan RR Barbaro RP Rycus PT Mcmullan DM Conrad SA Fortenberry JD. Extracorporeal life support organization registry international report 2016. ASAIO J. (2017) 63:60–7. 10.1097/MAT.0000000000000475 27984321

[4] Su Y Liu K Zheng J-L Li X Zhu D-M Zhang Y. Hemodynamic monitoring in patients with venoarterial extracorporeal membrane oxygenation. Ann Transl Med. (2020) 8:792. 10.21037/atm.2020.03.186 32647717 PMC7333156

[5] Hou JY Li X Yang SG Zheng JL MA JF Su Y. Veno-arterial extracorporeal membrane oxygenation for patients undergoing heart transplantation: A 7-year experience. Front Med. (2021) 8:774644. 10.3389/fmed.2021.774644 34988094 PMC8720851

[6] Hou JY Wang CS Lai H Sun YX Li X Zheng JL. Veno-arterial extracorporeal membrane oxygenation for patients undergoing acute type A aortic dissection surgery: A six-year experience. Front Cardiovasc Med. (2021) 8:652527. 10.3389/fcvm.2021.652527 34079828 PMC8165157

[7] Radsel P Goslar T Bunc M Ksela J Gorjup V Noc M. Emergency veno-arterial extracorporeal membrane oxygenation (VA ECMO)-supported percutaneous interventions in refractory cardiac arrest and profound cardiogenic shock. Resuscitation. (2021) 160:150–7. 10.1016/j.resuscitation.2020.11.028 33309699

[8] Wang L Yang F Zhang S Li C Du Z Rycus P. Percutaneous versus surgical cannulation for femoro-femoral VA-ECMO in patients with cardiogenic shock: Results from the Extracorporeal Life Support Organization Registry. J Heart Lung Transplant. (2022) 41:470–81. 10.1016/j.healun.2022.01.009 35125287

[9] Au SY Chan KS Fong KM Leung PWR Ng WYG So SO. One-year experience of bedside percutaneous VA-ECMO decannulation in a high-ECMO-volume center in Hong Kong. Perfusion. (2021) 36:803–7. 10.1177/0267659120971998 33200650

[10] Au S-Y Chan K-S Fong K-M Wong H-MR Fong Y-H Chui S-F. Comparing the outcomes of bedside percutaneous VA-ECMO decannulation by ProGlide and Manta in a high-ECMO-volume center in Hong Kong. Artif Organs. (2022) 46:1382–8. 10.1111/aor.14198 35132654

[11] Hassan MF Lawrence M Lee D Velazco J Martin C Reddy R. Simplified percutaneous VA ECMO decannulation using the MANTA vascular closure device: Initial US experience. J Card Surg. (2020) 35:217–21. 10.1111/jocs.14308 31899831

